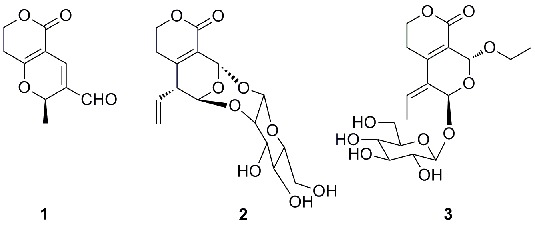# Anti-hepatitis B virus active secoiridoids from *Swertia kouitchensis*

**DOI:** 10.1007/s13659-011-0009-5

**Published:** 2011-09-05

**Authors:** Kang He, Yun-Bao Ma, Chang-An Geng, Xue-Mei Zhang, Tuan-Wu Cao, Fu-Qiang Jiang, Ji-Jun Chen

**Affiliations:** 1 State Key Laboratory of Phytochemistry and Plant Resources in West China, Kunming Institute of Botany, Chinese Academy of Sciences, Kunming, 650201 China; 2 Graduate University of Chinese Academy of Sciences, Beijing, 100039 China

**Keywords:** *Swertia kouitchensis*, anti-HBV activity, swertiakoulactone, swertiakoside A, swertiakoside B

## Abstract

Three new secoiridoids, swertiakoulactone (**1**) and swertiakosides A and B (**2** and **3**), were isolated from *Swertia kouitchensis*. Their structures were elucidated by comprehensive spectroscopic analyses including MS, IR, 1D and 2D NMR data. By the anti-hepatitis B virus (HBV) assay on Hep G 2.2.15 cells line *in vitro*, compound **1** showed moderate activities inhibiting the HBsAg secretion (IC_50_ = 1.10 *m*M, SI = 4.39) and HBV DNA replication (IC_50_ = 1.16 *m*M, SI = 4.12).

## Introduction

*Swertia kouitchensis* (Gentianaceae), is a perennial herb mainly distributed in Yunnan, Sichuan, and Guizhou provinces of China,[1] which was widely used as a fork medicine to treat jaundice, indigestion and sore throat.[2] Its congener plant *S. mileensis*, well-known as Qing-Ye-Dan in Chinese, has been documented in Chinese Pharmacopoeia (1977–2005 editions) for treatment of viral hepatitis.[3] Our previous investigation on *S. mileensis* resulted in a series of novel anti-HBV active secoiridoid dimers with $C_{18}$ and $C_{20}$ skeletons (swerilactones A and B[4], C and D[5]) and secoiridoid trimers with $C_{29}$ skeleton (swerilactones H–K)[6], as well as three unusual aromatic lactones (swerilactones E–G)[7] and three new secoiridoid glycoside dimers (swerilactosides A–C)[8]. Presently, xanthones, triterpenoids and sterols except for iridoids have been reported from *S. kouitchensis*.[9,10] As an on-going search for anti-HBV active compounds from natural resources, our investigation on *S. kouitchensis* led to the isolation of three new compounds, including one secoiridoid, swertiakoulactone (**1**), and two secoiridoid glycosides, swertiakosides A and B (**2** and **3**). Herein, we report the isolation, structural elucidation, and anti-HBV properties of compounds **1**–**3**.

## Results and Discussion

Swertiakoulactone (**1**) was obtained as amorphous solids. The positive HRESIMS spectrum showed the $[M + H]^+$ ion peak at *m/z* 195.0654 (calcd 195.0657) in accordance with the molecular formula $C_{10}H_{10}O_4$, indicating six degrees of unsaturation. The IR spectrum suggested the presence of C=O (1714, 1667 $cm^{-1}$) and C=C bonds (1639 $cm^{-1}$). The $^1H$ and $^{13}C$ NMR (DEPT) spectra (Table 1) displayed ten carbon resonances due to four quaternary carbons, three methines, two methylenes and one methyl group, of which one lactone, one aldehyde group and two double bonds were revealed. Besides the four degrees of unsaturation ascribed to two C=O and two C=C groups, the remaining two degrees of unsaturation required compound **1** to contain a dicyclic ring system. A $\delta$-lactone ring was characterized from the $^1H$ NMR [$\delta_H$ 2.71, (1H, dt, 18.2, 6.4), 2.66 (1H, dt, 18.2, 6.4), 4.44 (2H, t, $J$ = 6.4 Hz)]

**1** **2** **3**

and $^{13}C$ NMR [$\delta_C$ 163.8 (C-11), 101.1 (C-4), 171.6 (C-5)] data, which was supported by the $^1H$–$^1H$ COSY correlated signal of H-6/H-7 and HMBC correlations from H-7 to C-5 and C-11, and from H-6 to C-4 (Figure 1). In addition, a $\gamma$-pyran ring joined with the $\delta$-lactone at C-4 and C-5 positions was deduced from the $^1H$ NMR [$\delta_H$ 7.29, (1H, s), 5.60 (1H, q, $J$ = 6.5 Hz)] and $^{13}C$ NMR [$\delta_C$ 129.6 (C-2), 135.4 (C-3), 74.1 (C-9)] data, together with the HMBC experiment (Figure 1) (H-3/C-5, C-11; and H-9/C-3, C-5). The residual two carbons,

*To whom correspondence should be addressed. E-mail: chenjj@mail.kib.ac.cn.

ascribed as one methyl and one conjugated aldehyde groups, were assigned at C-9 and C-2 respectively, by the correlations of H-9/Me-10 in $^1$H–$^1$H COSY spectrum and H-1/C-3, C-9 in HMBC spectrum (Figure 1). Thus, the structure of swertiakoulactone (**1**) was assigned as shown.

COSY (**—**) HMBC (H→C)

**Figure 1.** The key 2D NMR correlations for **1**.

Swertiakoside A (**2**), amorphous solids, possessed the molecular formula $C_{16}H_{20}O_9$ deduced from negative ESIMS ([M − H]$^-$, *m/z* 355) and HRESIMS (*m/z* 355.1034, ([M − H]$^-$; calcd 355.1029)) analyses. The IR spectrum displayed the absorption bands for OH (3431 cm$^{-1}$), C=O (1708 cm$^{-1}$), C=C (1636 cm$^{-1}$), and C-O-C (1104, 1047 cm$^{-1}$) functionalities. The $^{13}$C NMR (DEPT) spectrum (Table 1) exhibited sixteen C atoms assignable to three quaternary carbons, nine methines and four methylenes. The observed carbon signals [$\delta_C$ 98.9 (C-1′), 81.6 (C-2′), 76.2 (C-3′), 70.7 (C-4′), 79.0 (C-5′), 62.5 (C-6′)] in the $^{13}$C NMR spectrum, in combination with the coupling constant ($J$ = 7.1 Hz) of the anomeric H atom ($\delta_H$ 4.97) in $^1$H NMR spectrum suggested the $\beta$-D-glucosyl moiety.[11] The NMR data of swertiakoside A (**2**) were similar to those of swertiajaposide A,[11] except for the absence of methoxyl group, as well as the downfield shift of C-2′ from $\delta_C$ 74.7 (d) in swertiajaposide A to 81.6 (d) in compound **2**. The

COSY (**—**) HMBC (H→C) ROESY (H↔H)

**Figure 2.** The key 2D NMR correlations for **2**.

HMBC correlations (Figure 2) of H-1 [$\delta_H$ 5.77 (d, $J$ = 2.0 Hz)] with C-2′, and H-1′ [$\delta_H$ 4.97 (d, $J$ = 7.1 Hz)] with C-3 indicated the linkage of C(1)–O–C(2′) and C(3)–O–C(1′). Therefore, the planar structure of compound **2** was constructed. In the ROESY spectrum (Figure 2), correlations of H-3 with H-1′ and H-9 indicated the same orientation of H-3, H-1′ and H-9. Based on the structure of **2** shown in figure 2, the anomeric H-atom (H-1′) was put at the upside of the reference plane ($\beta$-orientation), therefore, H-3 and H-9 were also determined as $\beta$-orientation. Similarly, the obviously correlated signal of H-1 with H-10 indicated the $\alpha$-orientation of H-1, which was consistent with the undetected correlation of H-1 with H-3. Hence, the structure of swertiakoside A (**2**) was established.

Swertiakoside B (**3**) was obtained as amorphous solids. Its molecular formula $C_{18}H_{26}O_{10}$ was determined by positive ESIMS ([M + Na]$^+$, *m/z* 425) and HRESIMS (*m/z* 425.1434, ([M + Na]$^+$; calcd 425.1423)). Acid hydrolysis of compound **3** with 10% $H_2SO_4$ yielded glucose, which was identified by comparison with an authentic sample on PC (see Experiment Part).[12] Comparison of its 1D NMR spectra (Table 1) with those of 3-*epi*-swertiajaposide C[13] showed high similarity. The major difference was that the methoxyl group in 3-*epi*-swertiajaposide C [$\delta_C$ 57.7 (t)] was replaced by an ethoxyl group in compound **3** [$\delta_C$ 67.5 (t), 15.2 (q)], which was confirmed by the $^1$H–$^1$H COSY correlation of H-12/H-13 and HMBC correlation from H-3 [$\delta_H$ 5.67 (s)] to C-12 [$\delta_C$ 67.5 (t)]

COSY (**—**) HMBC (H→C) ROESY (H↔H)

**Figure 3.** The key 2D NMR correlations for **3**.

(Figure 3). The relative configuration of **3** was deduced based on ROESY analyses and NMR comparison, which was identical to 3-*epi*-swertiajaposide C[13]. The ROESY cross-peak (Figure 3) between H-1 and Me-10 suggested the ($Z$)-configuration of C(8) = C(9) bond. Similarly, the glucosyl group at C-1 and the ethoxyl group at C-3 were proposed as *trans*-orientation based on the $^{13}$C NMR data [$\delta_C$ 90.9 (C-1), 94.4 (C-3)], as well as the undetected correlation of H-1 with H-3 in the ROESY spectrum.[13] Consequently, the structure of swertiakoside B (**3**) was elucidated as shown.

Compounds **1**–**3** were evaluated for their anti-HBV activities, namely inhibiting the secretion of hepatitis B surface antigen (HBsAg) and hepatitis B e antigen (HBeAg), as well as HBV DNA replication in Hep G 2.2.15 cells, as reported previously (tenofovir was used as the positive control).[6] The results showed that compound **1** exhibited moderate activity inhibiting the secretion of HBsAg with an $IC_{50}$ value of 1.10 *m*M (SI = 4.39) and HBV DNA replication with an $IC_{50}$ value of 1.16 *m*M (SI = 4.12). However, compounds **2** and **3** showed no anti-HBV activity and cytotoxicity at the highest tested concentration (2.86 *m*M and 1.73 *m*M, respectively).

## Experimental Section

**General Experimental Procedures.** Column chromatography (CC): silica gel ($SiO_2$; 200–300 mesh, Qingdao Makall Chemical Co. Ltd., Qingdao, P. R. China), Sephadex LH-20 (Pharmacia, Fine Chemical Co. Ltd. Uppsala, Sweden), and Lichroprep RP-18 (40–63 μm; Merck, D-Darmstadt). Fractions were monitored by TLC, visualizing by spraying with 10% $H_2SO_4$ in EtOH followed by heating. Optical rotations: Jasco model 1020 polarimeter (Horiba, Tokyo, Japan). UV Spectra: UV-210A spectrometer (Shimadzu, Kyoto, Japan); $\lambda_{max}$ (log $\varepsilon$) in nm. IR Spectra: Bio-Rad FTS-135 spectrometer (Bio-Rad, Hercules, California, USA); KBr pellets; in cm$^{-1}$. $^1$H and $^{13}$C NMR spectra: Bruker DRX-500 or avance III-600

spectrometers (Bruker, Bremerhaven, Germany); with TMS as internal standard; $\delta$ in ppm, J in Hz. ESI and HRESIMS: VG Auto Spec-3000 spectrometer (VG, Manchester, UK); in m/z (rel. %).

**Plant Material.** The whole plant of *S. kouitchensis* was collected in Jianshui County, Yunnan Province, China, in December 2008, and authenticated by Prof. Li-Gong Lei, Kunming Institute of Botany, Chinese Academy of Sciences. A voucher specimen (No. 2008-12-12) has been deposited in the Laboratory of Antivirus and Natural Medicinal Chemistry, Kunming Institute of Botany, Chinese Academy of Sciences.

**Table 1.** $^{1}$**H and** $^{13}$**C NMR data for 1–3.**

| Pos. | **1**[a] | | **2**[b] | | **3**[b] | |
|---|---|---|---|---|---|---|
| | $\delta_H$ (*J* in Hz) | $\delta_C$ | $\delta_H$ (*J* in Hz) | $\delta_C$ | $\delta_H$ (*J* in Hz) | $\delta_C$ |
| 1 | 9.46, s | 189.4, CH | 5.77, d (2.0) | 91.3, CH | 6.23, s | 90.9, CH |
| 2 | | 129.6, C | | | | |
| 3 | 7.29, s | 135.4, CH | 5.35, s | 95.4, CH | 5.67, s | 94.4, CH |
| 4 | | 101.1, C | | 121.3, C | | 119.8, C |
| 5 | | 171.6, C | | 158.7, C | | 147.1, C |
| 6a | 2.71, dt (18.2, 6.4) | 27.5, $CH_2$ | 2.74, m | 28.7, $CH_2$ | 2.76, m | 23.8, $CH_2$ |
| 6b | 2.66, dt (18.2, 6.4) | | 2.42, m | | 1.26, m | |
| 7a | 4.44, t (6.4) | 63.6, $CH_2$ | 4.45, dd (8.6, 3.8) | 66.7, $CH_2$ | 4.48, m | 66.7, $CH_2$ |
| 7b | | | | | 4.37, m | |
| 8 | | | 5.68, m | 132.8, CH | 6.41, q (14.5, 7.2) | 136.6, CH |
| 9 | 5.60, q (6.5) | 74.1, CH | 3.13, br d (8.4) | 50.7, CH | | 130.6, C |
| 10 | 1.44, d (6.5) | 20.6, $CH_3$ | 5.31, m | 121.5, $CH_2$ | 1.97, d (7.2) | 14.3, $CH_3$ |
| 11 | | 163.8, C | | 163.9, C | | 165.6, C |
| 12a | | | | | 4.07, m | 67.5, $CH_2$ |
| 12b | | | | | 3.73, m | |
| 13 | | | | | 1.25, t (7.1) | 15.2, $CH_3$ |
| 1′ | | | 4.97, d (7.1) | 98.9, CH | 4.83, d (7.9) | 99.0, CH |
| 2′ | | | 3.33, m | 81.6, CH | 3.21, dd (9.0, 7.9) | 75.0, CH |
| 3′ | | | 3.55, t (8.9) | 76.2, CH | 3.44, t (9.0) | 78.1, CH |
| 4′ | | | 3.31, m | 70.7, CH | 3.29, m | 71.7, CH |
| 5′ | | | 3.34, m | 79.0, CH | 3.38, m | 78.5, CH |
| 6′a | | | 3.86, dd (12.0, 1.9) | 62.5, $CH_2$ | 3.92, dd (11.5, 1.8) | 63.0, $CH_2$ |
| 6′b | | | 3.65, dd (12.0, 5.6) | | 3.68, dd (11.5, 6.1) | |

[a]Recorded in $CDCl_3$ at 600 MHz; [b]Recorded in $CD_3OD$ at 500 MHz.

**Extraction and Isolation.** The whole plant of *S. kouitchensis* (1.5 kg) was powered and successively extracted with 90% and 50% EtOH under reflux for 2 times (each 5 L, 2 h). The extracts were combined, and evaporated under reduced pressure. The crude residue was suspended in $H_2O$, and partitioned with petroleum ether, EtOAc, and *n*-butanol successively. The EtOAc part (72 g) was subjected to a $SiO_2$ CC (700g, 200−300 mesh) and eluted with gradient $CHCl_3/MeOH/H_2O$ (100:0:0, 95:5:0, 90:10:0, 80:20:2, 70:30:3) to yield six fractions Frs. A-F. Fr. B (6.6 g) was subjected to a $SiO_2$ CC with a gradient elution of $CHCl_3/Me_2CO$ (from 9:1 to 5:5) to supply Frs. B1–B3. Fr. B3 (1.4 g) was chromatographed on a $SiO_2$ CC eluted with $PE/Me_2CO$ (7:3) and then purified on Sephadex LH-20 (MeOH), yielding **1** (7 mg). Fr. E (2.2 g) was subjected by RP-18 CC ($MeOH/H_2O$ 2:8), and subsequently performed on $SiO_2$ CC with $CHCl_3$/MeOH (9:1–8:2) and purified by Sephadex LH-20 (MeOH) to obtain **2** (20 mg) and **3** (25 mg).

**Swertiakoulactone (1):** amorphous solids; $[\alpha]_D^{20} = -4.9$ (*c* 0.27, $CHCl_3$); UV ($CHCl_3$) $\lambda_{max}$ (log $\varepsilon$) 338 (3.6), 261 (3.8) nm; IR (KBr) $\nu_{max}$ 2919, 2850, 1714, 1667, 1639, 1571, 1427, 1168 $cm^{-1}$; NMR data, see Table 1; ESIMS *m/z* 195 ($[M + H]^+$); positive ion HRESIMS *m/z* 195.0654 (calcd for $C_{10}H_{11}O_4$ $[M + H]^+$, 195.0657).

**Swertiakoside A (2):** amorphous solids; $[\alpha]_D^{20} = -55.6$ (*c* 0.29, MeOH); UV (MeOH) $\lambda_{max}$ (log $\varepsilon$) 265 (3.4) nm; IR (KBr) $\nu_{max}$ 3431, 1708, 1636, 1104, 1047 $cm^{-1}$; NMR data, see Table 1; ESIMS *m/z* 355 ($[M - H]^-$); negative ion HRESIMS *m/z* 355.1034 (calcd for $C_{16}H_{19}O_9$ $[M - H]^-$, 355.1029).

**Swertiakoside B (3):** amorphous solids; $[\alpha]_D^{20} = -53.0$ (*c* 0.08, MeOH); UV (MeOH) $\lambda_{max}$ (log $\varepsilon$) 272 (4.0), 202(3.7) nm; IR (KBr) $\nu_{max}$ 3440, 1631, 1075, 1059 $cm^{-1}$; NMR data, see Table 1; ESIMS *m/z* 425 ($[M + Na]^+$); positive ion HRESIMS *m/z* 425.1434 (calcd for $C_{18}H_{26}NaO_{10}$ $[M + Na]^+$, 425.1423).

**Acid Hydrolysis of Swertiakoside B (3).** A solution of **3** (3 mg) in a mixture of MeOH (1.0 mL) and 10% $H_2SO_4$ (1.0 mL) was refluxed for 2 h. The hydrolysate was allowed to cool, diluted with 2 mL of $H_2O$, and extracted with 4 mL of AcOEt for 3 times. The aq. layer was neutralized with aq. $Ba(OH)_2$ and concentrated in vacuum to give a residue, in which glucose (from **3**) was identified by comparison with the authentic sample ($BuOH/AcOEt/H_2O$ 4:1:5, upper layer, $R_f$ 0.45; $PhOH/H_2O$ 4:1, $R_f$ 0.50) on PC: visualized by spraying with phthalic acid/aniline reagent (1.66 g phthalic acid and 0.93 g aniline dissolved in 100 mL $H_2O$/sat. BuOH), followed by heating.

**In Vitro Anti-HBV Assay.** The anti-HBV procedure was performed according to our previous report.[8] Briefly, compounds **1**–**3** were evaluated in the Hep G 2.2.15 cell line, which was stably transfected (Lipofectamine 2000 reagent; Invitrogen; Carlsbad, CA, USA) with the HBV genome. The toxicity of the compounds was assayed by using a modified MTT (GIBCO Invitrogen, Carlsbad, CA, USA) method. DMSO (Gibco; solvent control) alone was added to each culture as a solvent control. All the compounds evaluated were dissolved in DMSO for the assays of anti-HBV activity and cytotoxicity. The concentration of DMSO in the culture was kept below 2.5 $\mu$L $mL^{-1}$ to ensure that the growth of the cells was not affected.

**Cell Line and Cell Culture.** The widely used Hep G 2.2.15 cell line was applied for the assay of anti-HBV activities. In this study, Hep G 2.2.15 cells established from a hepatoma cell line Hep G 2 (ATCC, Manassas, VA, USA) were cultured in RPMI-1640 (GIBCO) medium supplemented with 10% fetal calf serum (GIBCO), 100 $\mu$g $mL^{-1}$ G148 (GIBCO), 100 IU/mL penicillin (GIBCO), and 100 IU/mL streptomycin (GIBCO). All cultures were maintained at 37°C in a moist atmosphere containing 5% $CO_2$.

**Analysis of Secreted HBV Antigens.** The sub-toxic concentration of the identified compounds was measured with a serial dilution in 96-well microplates in which cells were seeded at a density of $3\times10^4$ cells/mL and cultured at 37 °C, 5% $CO_2$ for 12 days. After incubation, the cells and supernatants were collected. The levels of HBsAg and HBeAg in the supernatants were assayed with an ELISA (AutoBio Diagnostics Co., Ltd., Beijing, P. R. of China) method. The absorbance (A) of each well was measured at 490 nm using a microplate reader (Model 680, Bio-Rad, Inc., Hercules, CA, USA).

**Determination of HBV DNA Replication.** HepG 2.2.15 cells were seeded in 24 well culture plates at a density of $5 \times 10^5$ cells per well. Every 2 days, medium was changed. After 6 days, compounds were added to the cell cultures, and fresh medium was fed every other day for another 6 days. Cells were collected and total DNA was isolated by using TIANamp Gemomic DNA Kit (TIANGEN, Biotech Co., Ltd, China) following the manufacturer's instructions. For detection of HBV DNA, a real-time PCR assay was used.

**Cytotoxicity Assay.** The toxicity of compounds **1**–**3** was assayed by a modified MTT method. In brief, the samples were prepared at different concentrations. After Hep G 2.2.15 cells had been seeded in a 96-well microplate for 4 h, the samples (20 mL) were placed in each well and incubated for 3 days at 37°C; then, 0.1 mL MTT [3-(4,5-dimethylthiazole-2-yl)-2,5-diphenyltetrazolium bromide, 400 mg $mL^{-1}$] was added for 4 h. After removal of MTT medium, DMSO (100 mL/well) was added into the microplate for 10 min. The formazan crystals were dissolved, and the absorbance was measured on a microplate reader at 490 nm.

**Acknowledgments**

This work was financially supported by the National Natural Sciences Foundation of China for Distinguished Young Scholar (No. 81025023). The authors are grateful to the staff of the analytical group of the State Key Laboratory of Phytochemistry and Plant Resources in West China, Kunming Institute of Botany, Chinese Academy of Sciences, for the measurements of all spectra.

**Open Access** This article is distributed under the terms of the Creative Commons Attribution License which permits any use, distribution, and reproduction in any medium, provided the original author(s) and source are credited.
